# KCTD15 is overexpressed in human childhood B-cell acute lymphoid leukemia

**DOI:** 10.1038/s41598-019-56701-7

**Published:** 2019-12-27

**Authors:** Giovanni Smaldone, Giuliana Beneduce, Mariarosaria Incoronato, Katia Pane, Monica Franzese, Luigi Coppola, Angela Cordella, Rosanna Parasole, Mimmo Ripaldi, Giovanni Nassa, Andrea Soricelli, Luigi Vitagliano, Peppino Mirabelli, Marco Salvatore

**Affiliations:** 1 IRCCS SDN, Via E. Gianturco 113, 80143 Napoli, Italy; 2 Laboratory of Molecular Medicine and Genomics, Department of Medicine and Surgery, University of Salerno, Baronissi, Italy; 3 Institute of Biostructures and Bioimaging, C.N.R., 80134 Napoli, Italy; 4 Department of Pediatric Hematology-Oncology, Santobono-Pausilipon Hospital, Naples, Italy; 5 Department of Sport Sciences & Healthiness, University of Naples ‘Parthenope’, 80131 Napoli, Italy

**Keywords:** Diagnostic markers, Acute lymphocytic leukaemia

## Abstract

Leukemic cells originate from the malignant transformation of undifferentiated myeloid/lymphoid hematopoietic progenitors normally residing in bone marrow. As the precise molecular mechanisms underlying this heterogeneous disease are yet to be disclosed, the identification and the validation of novel actors in leukemia is of extreme importance. Here, we show that KCTD15, a member of the emerging class of KCTD ((K)potassium Channel Tetramerization Domain containing) proteins, is strongly upregulated in patients affected by B-cell type acute lymphoblastic leukemia (B-ALL) and in continuous cell lines (RS4;11, REH, TOM-1, SEM) derived from this form of childhood leukemia. Interestingly, KCTD15 downregulation induces apoptosis and cell death suggesting that it has a role in cellular homeostasis and proliferation. In addition, stimulation of normal lymphocytes with the pokeweed mitogen leads to increased KCTD15 levels in a fashion comparable to those observed in proliferating leukemic cells. In this way, the role of KCTD15 is likely not confined to the B-ALL pathological state and extends to activation and proliferation of normal lymphocytes. Collectively, data here presented indicate that KCTD15 is an important and hitherto unidentified player in childhood lymphoid leukemia, and its study could open a new scenario for the identification of altered and still unknown molecular pathways in leukemia.

## Introduction

Leukemic cells originate from the malignant transformation of undifferentiated myeloid or lymphoid hematopoietic progenitors normally residing in bone marrow^1^. The different types of leukemias are caused by either genetic or environmental alterations^1–6^, although the precise molecular mechanisms underlying this heterogeneous disease are yet to be disclosed. Acute lymphoid leukemia (ALL) represents more than three-quarters of all childhood leukemias, with the precursor B-cell type (B-ALL) being the most common form (approximately 80% of ALL)^7,8^. Despite the successes in therapeutic treatments achieved in the last decades, B-ALL continues to be the principal cause of cancer deaths in children and it is still considered a therapeutic challenge for pediatric oncologists^9,10^. Therefore, more efforts are needed to better define the process of leukemogenesis and, consequently, identify novel disease biomarkers and/or therapies for clinical management.

The origin of leukemic cells resides in the malignant transformation of undifferentiated myeloid or lymphoid hematopoietic progenitors normally residing in bone marrow (BM). Consequently, the classification of ALL is performed according to their immunological profile^1^. In this context, multiparameter Flow CytoMetry (FCM) is efficiently used to have a rapid and effective mean for disease diagnosis and classification. The immunological classification of leukemia is necessary also for having prognostic information and a modality for minimal residual disease evaluation^3,4^. However, it is important to consider that B-ALL comprises multiple subtypes also featured by chromosomal abnormalities such as translocations able to give rise to chimeric fusion genes or different levels of aneuploidy. Furthermore, it is important to consider the occurrence of additional aberrations that are able to contribute to leukemogenesis as in the case of deletions, amplifications, sequence mutations, and epigenetic lesions^5^. In this intricate scenario, the identification of important players, at genetic and protein levels, may represent a valuable tool to gain insights into the mechanism underlying the etiology of the disease. Moreover, the identification and validation of novel actors in ALL are important to increase the current repertoire of biomarkers available for the clinical management of this disease^5^.

On the basis of these considerations, we here started a search for novel leukemia biomarkers by comparing the RNA expression profiles of B-ALL patients with those of healthy subjects. These analyses highlighted that mRNAs of the gene encoding the protein KCTD15, a member of the emerging protein class KCTD (potassium channel tetramerization domain)^11–16^, is strongly upregulated in common B-ALL patients. This finding was also confirmed at the protein level in both human B-ALL cell lines and patients. The effects produced by the down- and the up-regulation of KCTD15 were also monitored using leukemic and normal cells. Collectively, data here presented indicate that KCTD15 is an important and hitherto unidentified player in this kind of childhood leukemia.

## Results

### KCTD15 expression in common B-ALL and after induction therapy

**Figure 1:**
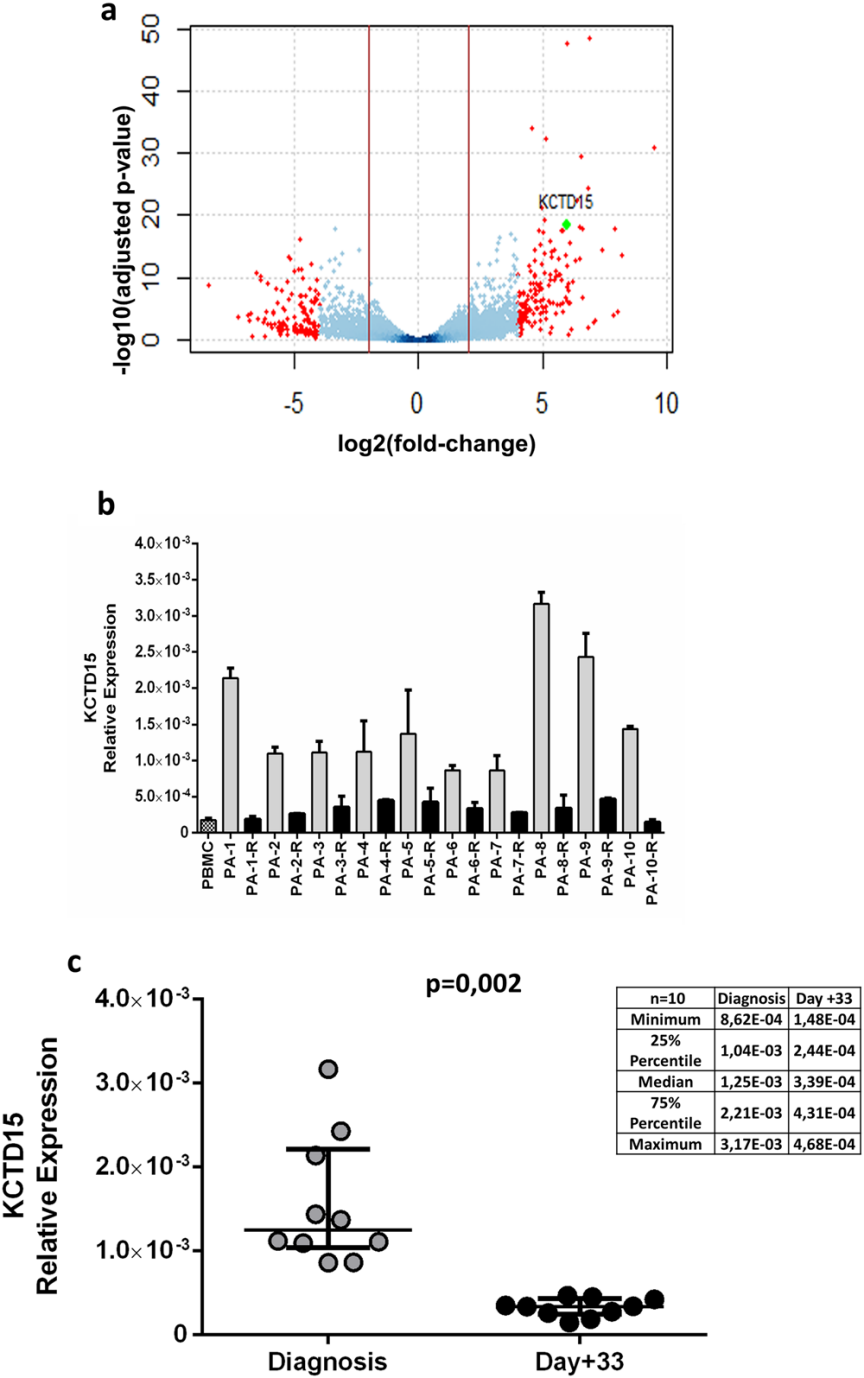
KCTD15 is up-regulated in common B-cell acute leukemia. **(a**) Volcano plot displaying 16976 filtered genes between peripheral blood B cells from 3 healthy subjects and leukemic cells from 3 patients affected by B-ALL. The adjusted p-value is calculated by the Benjamini and Hochberg’s algorithm. Red dots represent differential expressed genes; these are in total 683 genes identified from the overlap of two different statistical methods (NoiSeqBio and DESeq. 2) with a threshold (red vertical line) at |log2FC| ≥ 2; the green dot highlights KCTD15 gene. (**b**) KCTD15 mRNA expression levels in a group of 10 common B-ALL patients at diagnosis (coded as PA-#) and day +33 after therapy (coded as PA-#-R). The relative expression was determined using the 2^−ΔCt^ method. KCTD15 relative expression is shown as mean +/− SD of two technical independent experiments. (**c**) KCTD15 levels were plotted according to the relative expression measured in BM cells from patients at diagnosis (grey circles) and after therapy (black circles) at day + 33. Transcription of KCTD15 significantly decreased from a median value of 1.25 × 10^−3^ to 3.38 × 10^−4^. (**p = 0.002, Wilcoxon matched-pairs test). The inset reports the statistical parameters for the two ensembles.

**Figure 2:**
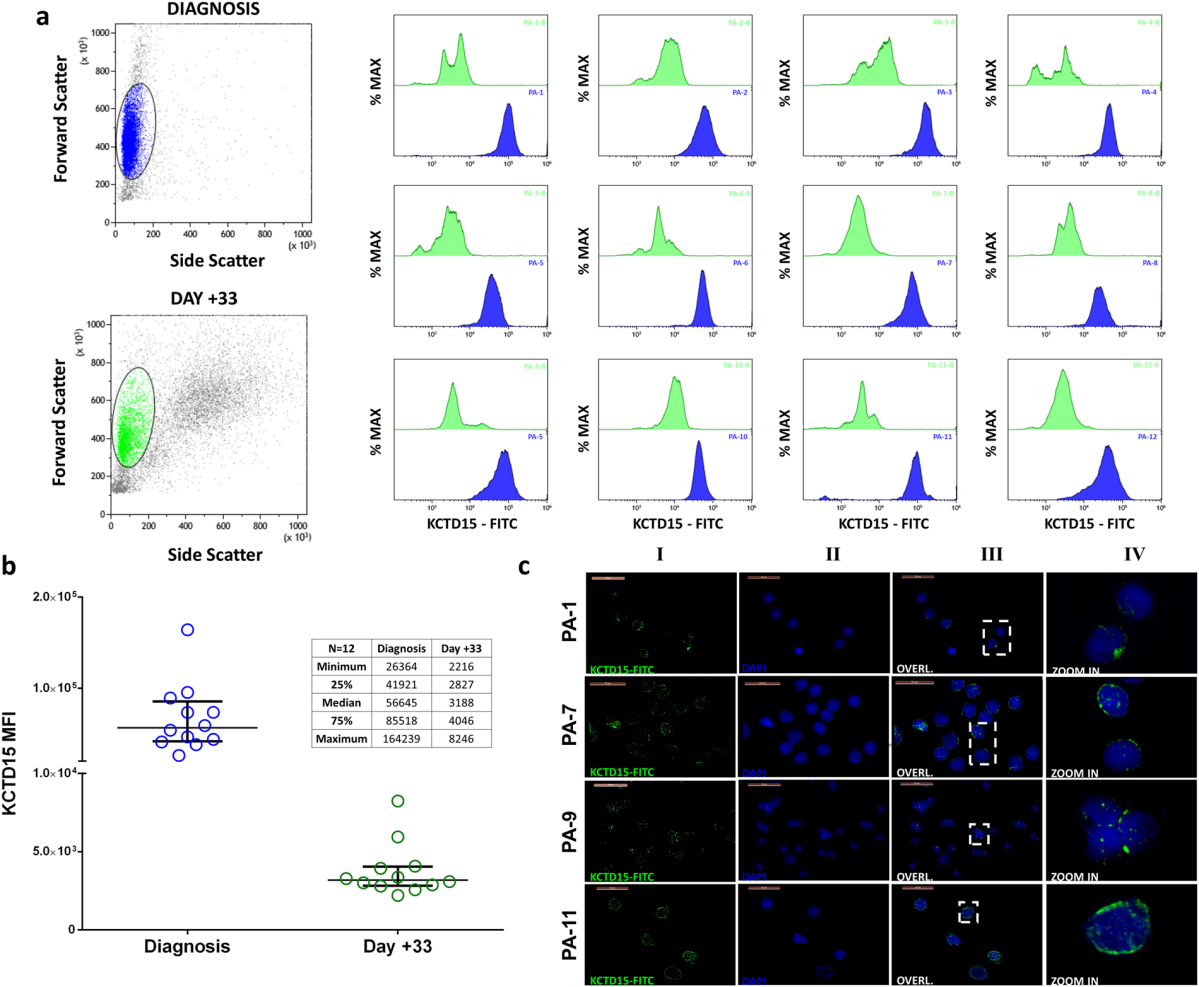
KCTD15 expression in pediatric B-ALL patients. (**a**) KCTD15 intracellular expression measured by mean fluorescence intensity (MFI) for the twelve patients at diagnosis (blue histograms-Patients coded as PA-#) and at day +33 (green histograms- Patients coded as PA-#-R) after therapy. Forward Scatter vs SideScatter plot on the left represent cellular gating strategy. (**b**) KCTD15 protein levels were measured by FCM for each patient at diagnosis (blue circles) and after therapy (green circles) at day +33. Protein levels significantly decreased from a median value of 5.66*10^4^ to 3.1*10^3^ MFI (***p = 0.0005, Wilcoxon matched-pairs test). The inset reports the statistical parameters for the two ensembles. (**c**) Fluorescence microscopy experiments of bone marrow smears from four consecutive B-ALL patients. Endogenous KCTD15 was labeled with FITC-conjugated secondary antibody. Column I) KCTD15-FITC fluorescence (green). Column II) Nuclei staining with DAPI (blue). III) Overlapping of FITC and DAPI channels. IV) Enlarged detail of overlapped channels. PA-# = patient-# at diagnosis. Magnification 63 × . Scale bars 20 µm.

**Figure 3:**
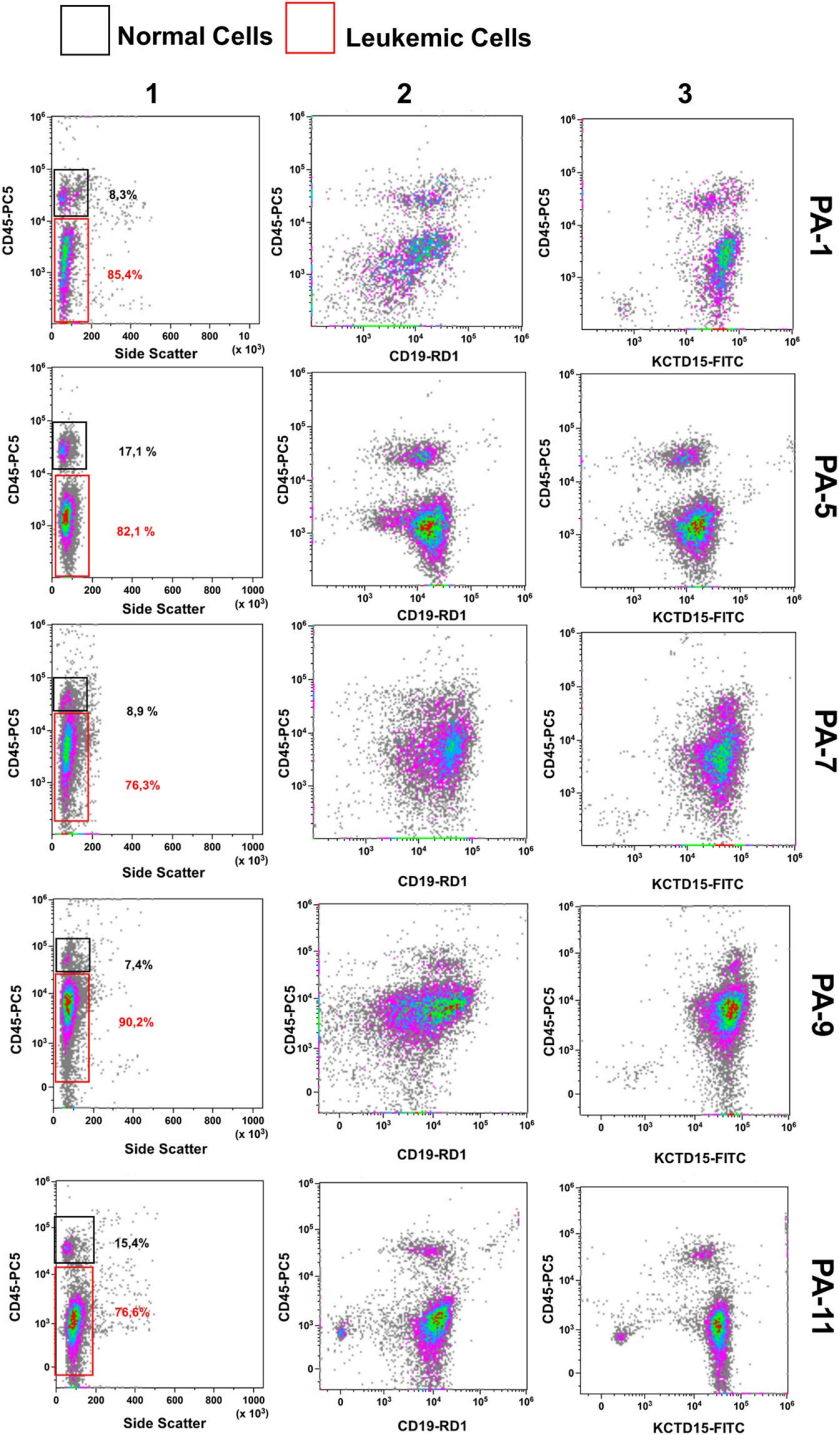
Multiparametric FlowCytoMetry (FCM) analysis of pediatric B-ALL bone marrow. Surface (for CD45 and CD19) and intracellular (KCTD15) staining are reported for 5 exemplificative cases of pediatric B-ALL BM samples. Leukemia B-cells (red rectangle) are immunophenotypically detectable by means of intermediate/low expression of CD45 antigen (Column 1) and intermediate expression of CD19 (Column 2 - CD45^dim^/CD19^dim^). Normal resting lymphocytes (black rectangle) display bright CD45 expression and low CD19 intensity (CD45^bright^/CD19^dim^). KCTD15 is brightly expressed in both types of cells (Column 3). PA-# = Patient-# at diagnosis.

The search for novel key players in leukemia etiology and/or for novel biomarkers was here started through gene expression profiling using RNA-sequencing (RNA-seq) of leukemic cells purified from three pediatric patients affected by B-ALL (Table 1, patients a-c). This profile was compared with that observed in naïve (mature) B cells from the peripheral blood of three healthy subjects (3 males with a median age of 36). A total of 25 out of 873 differentially expressed (DE) genes (Table 2) were found highly up-regulated in terms of fold change (Log2FC ≥ 6). Notably, gene-disease association analysis performed using the DisGeNET (v6.0) database^17^, MedLine and Ensembl indicate that 11 out of these 25 up-regulated genes have not been hitherto associated with leukemia (Table 2). Among these, we focused our attention on *KCTD15* being it one of the most up-regulated DE genes (Log2FC~6 and p-adj < 3.74e-19) as shown in the volcano plot reported in Fig. 1a. In order to confirm and extend the data emerged from the RNA-seq analysis, we decided to evaluate the expression of the *KCTD15* gene and of the encoded protein in a second set composed of 12 patients (patients 1–12 listed in Table 1) at diagnosis and at day + 33 after induction therapy when minimal residual disease was undetectable according to the clinical reports (data not shown) and bone marrow (BM) smear examination (Supplementary Fig. 1 for some exemplificative images). Real-time PCR, which was performed on 10 out of these 12 patients due to the low cellular amount available for 2 of them, clearly indicates that *KCTD15* transcription levels were significantly (p = 0.002, Wilcoxon Matched Pairs-test) higher (3.6 times) at diagnosis when compared to day + 33 after therapy (Fig. 1b,c). This finding was confirmed by the analysis of intracellular KCTD15 protein levels monitored using FCM (Fig. 2a). We found that KCTD15 expression was significantly lower (p = 0.0005, Wilcoxon Matched Pairs-test) after therapy (at day + 33) when compared to the counterpart at diagnosis (Fig. 2b). The detection of KCTD15 up-regulation in common B-ALL cells prompted us to determine its localization by fluorescence microscopy experiments (Fig. 2c and Supplementary Fig. 4). In line with data available for homologous KCTD proteins^18,19^, we found that KCTD15 is localized in the cytoplasm of leukemic cells. Furthermore, to test the possibility to discriminate leukemic and residual normal cell residing in the same patient BM samples on the basis of KCTD15 levels, we performed a multicolor FCM experiment with simultaneous detection of surface (CD45 and CD19) and intracellular KCTD15 on 5 cryopreserved BM mononuclear cells samples (Fig. 3). Interestingly, the KCTD15 expression pattern was similar to that exhibited by the CD19 antigen (Fig. 3) thus confirming its close association with leukemic B cells.

**Table 1:** Clinical and biological characteristics of fifteen patients (nine female and six male - mean age 6 years) with a diagnosis of B acute lymphoblastic leukemia enrolled at Santobono-Pausilipon Hospital and used in this study.

ID PATIENTS	Patient a	Patient b	Patient c	Patient 1	Patient 2
Age (years)	2	4,5	2,8	2,1	3,6
Sex	F	M	F	F	M
Race	Caucasian	Caucasian	Caucasian	Caucasian	Caucasian
White blood count	11320/mmc	1760/mmc	291600/mmc	89830/mmc	67550/mmc
Bone marrow blast (%)	70	80	70	90	68
Cytogenetic	46, XX	46, XY	46, XX	46, XX, del(11q23) [6]/46, XX, [4]	45, XY, i (9)(q10), -13, add(19)(p13)[8]/46, xy[8]
Molecoular biology	Negative	t (12;21) TEL/AML1 rearrangement	Negative	t(9;11) MLL/AF9 rearrangement	t (1;19) E2A/PBX1 rearrangement
Immunophenotype	Common B-ALL	Common B-ALL	Common B-ALL	Pro-B	B common
GPR day +8	Yes	Yes	Yes	Yes	Yes
BM blast <10% day +15	Yes	Yes	Yes	No	Yes
NCI risk	Standard	Standard	Standard	High	High
MRD risk	Intermediate	Intermediate	Intermediate	Intermediate	Standard
Complete remission	Yes	Yes	Yes	Yes	Yes
**ID PATIENTS**	**Patient 3**	**Patient 4**	**Patient 5**	**Patient 6**	**Patient 7**
Age (years)	11,1	5,3	1,5	10,9	3,3
Sex	M	M	M	F	F
Race	Caucasian	Caucasian	Caucasian	Caucasian	Caucasian
White blood count	3790/mmc	3800/mmc	6570/mmc	4730/mmc	4720/mmc
Bone marrow blast (%)	65	70	80	89	93
Cytogenetic	59, XXXY, +4, +4, +5, +6, +10 + 14, +17, 18, +21, +21[5]/46, XY, [10]	NE	52, XXY, +6, +14, +17, +21[4]/46, XY[11]	NE	NE
Molecoular biology	Negative	Negative	Negative	Negative	t(12;21) TEL/AML1 rearrangement
Immunophenotype	B common	B common	B common	B common	B common
GPR day +8	Yes	Yes	Yes	Yes	Yes
BM blast <10% day +15	Yes	Yes	Yes	Yes	Yes
NCI risk	High	Standard	Standard	High	Standard
MRD risk	Standard	Intermediate	Intermediate	Intermediate	Intermediate
Complete remission	Yes	Yes	Yes	Yes	Yes
**ID PATIENTS**	**Patient 8**	**Patient 9**	**Patient 10**	**Patient 11**	**Patient 12**
Age (years)	10,2	9,9	14,8	5,6	3
Sex	M	F	M	M	F
Race	Indian	Caucasian	Caucasian	Caucasian	Caucasian
White blood count	7490/mmc	120690/mmc	453600/mmc	4800/mmc	11540/mmc
Bone marrow blast (%)	80	90	95	70	80
Cytogenetic	NE	NE	NE	NE	NE
Molecoular biology	Negative	Negative	Negative	t(12;21) TEL/AML1 Rearrangement	Negative
Immunophenotype	B common	B Ph-like	B common	B common	B common
GPR day + 8	Yes	No	Yes	Yes	Yes
BM blast < 10% day + 15	Yes	No	Yes	Yes	Yes
NCI risk	High	High	High	Standard	High
MRD risk	Intermediate	NA	NA	Intermediate	NA
Complete remission	Yes	Yes	Yes	Yes	Yes

For each patient are reported: the epidemiological features, the disease characteristics at diagnosis (white blood count, bone marrow blast percentage; cytogeneic, molecular biology and immunophenotype), the response to treatment on day 8, the count blasts at day 15; and the stratification risk. Patients a-c were used for the RNAseq analysis. Patients 1–12 were used for the validation experiments. WBC: White Blood Count BM: Bone Marrow; GPR: Good Prednisone Responder; NCI: National Cancer Institute; MRD: Minimal Residual Disease; ALL acute lymphoblastic leukemia; NA = Not available.

**Table 2:** List of the top 25 up-regulated genes identified by the RNA-seq analysis.

Gene ID	Gene symbol	Gene Name	log2FoldChange	pvalue	padj	GOTERM_BP_DIRECT
ENSG00000196549	MME	membrane metalloendopeptidase(MME)	6,85828	1,96E-53	3,20E-49	GO:0001822~kidney development
ENSG00000130508	PXDN	peroxidasin(PXDN)	9,497178	3,65E-35	1,19E-31	GO:0001960~negative regulation of cytokine-mediated signaling pathway
ENSG00000148498	PARD3	par-3 family cell polarity regulator(PARD3)	6,524529	1,19E-33	3,23E-30	GO:0006461~protein complex assembly
**ENSG00000143344**	**RGL1**	**ral guanine nucleotide dissociation stimulator like 1(RGL1)**	**6**,**807473**	**2**,**10E-28**	**4**,**89E-25**	**GO:0007264~small GTPase mediated signal transduction**
**ENSG00000143434**	**SEMA6C**	**semaphorin 6C(SEMA6C)**	**6**,**377528**	**2**,**74E-26**	**5**,**59E-23**	**GO:0001755~neural crest cell migration**
**ENSG00000153885**	**KCTD15**	**potassium channel tetramerization domain containing 15**	**5**,**637092093**	**5**,**67E-22**	**3**,**74E-19**	**GO:0007275 multicellular organism development**
**ENSG00000140848**	**CPNE2**	**copine 2(CPNE2)**	**6**,**48559**	**4**,**60E-22**	**6**,**26E-19**	**GO:0071277~cellular response to calcium ion**
ENSG00000148773	MKI67	marker of proliferation Ki-67(MKI67)	6,602699	1,32E-21	1,54E-18	GO:0006259~DNA metabolic process
ENSG00000166349	RAG1	recombination activating 1(RAG1)	7,911977	1,83E-21	1,99E-18	GO:0002250~adaptive immune response
**ENSG00000052795**	**FNIP2**	**folliculin interacting protein 2(FNIP2)**	**6**,**073717**	**3**,**22E-19**	**2**,**02E-16**	**GO:0000122~negative regulation of transcription from RNA polymerase II promoter**
ENSG00000122025	FLT3	fms related tyrosine kinase 3(FLT3)	7,383095	9,39E-18	4,79E-15	GO:0001776~leukocyte homeostasis
ENSG00000120833	SOCS2	suppressor of cytokine signaling 2(SOCS2)	8,196046	7,26E-17	3,12E-14	GO:0001558~regulation of cell growth
ENSG00000157557	ETS2	ETS proto-oncogene 2, transcription factor(ETS2)	6,456807	1,92E-16	7,26E-14	GO:0000122~negative regulation of transcription from RNA polymerase II promoter
ENSG00000171105	INSR	insulin receptor(INSR)	6,295734	6,22E-16	2,07E-13	GO:0000187~activation of MAPK activity
**ENSG00000065534**	**MYLK**	**myosin light chain kinase(MYLK)**	**5**,**980766**	**4**,**05E-15**	**1**,**18E-12**	**GO:0006468~protein phosphorylation**
**ENSG00000205336**	**ADGRG1**	**adhesion G protein-coupled receptor G1(ADGRG1)**	**6**,**192801**	**3**,**03E-12**	**5**,**37E-10**	**GO:0001525~angiogenesis**
ENSG00000134531	EMP1	epithelial membrane protein 1(EMP1)	6,015907	5,47E-12	9,01E-10	GO:0007275~multicellular organism development
**ENSG00000273983**	**HIST1H3G**	**histone cluster 1 H3 family member g(HIST1H3G)**	**6**,**045191**	**1**,**34E-10**	**1**,**68E-08**	**GO:0000183~chromatin silencing at rDNA**
ENSG00000161940	BCL6B	B-cell CLL/lymphoma 6B(BCL6B)	6,588191	1,67E-09	1.68E-07	GO:0000122~negative regulation of transcription from RNA polymerase II promoter
ENSG00000152192	POU4F1	POU class 4 homeobox 1(POU4F1)	6,015401	1,43E-08	1.14E-06	GO:0000122~negative regulation of transcription from RNA polymerase II promoter
**ENSG00000226674**	**TEX41**	**testis expressed 41**	**6**,**094701**	**1**,**49E-08**	**1**.**18E-06**	**GO: Not assigned**
ENSG00000189060	H1F0	H1 histone family member 0(H1F0)	7,977572	7.86E-07	3.69E-05	GO:0006309~apoptotic DNA fragmentation
**ENSG00000153531**	**ADPRHL1**	**ADP-ribosylhydrolase like 1(ADPRHL1)**	**7**,**814898**	**2**.**86E-06**	**1**.**09E-04**	**GO:0006886~intracellular protein transport**
ENSG00000131016	AKAP12	A-kinase anchoring protein 12(AKAP12)	7,025709	1.19E-04	2.346E-03	GO:0006605~protein targeting
**ENSG00000166073**	**GPR176**	**G protein-coupled receptor 176(GPR176)**	**6**,**797679**	**0**,**000621**	**0**,**008943**	**GO:0007186~G-protein coupled receptor signaling pathway**

For each gene the p-value and the p-value adjusted (p-adj) for multiple testing calculated by the Benjamini and Hochberg’s algorithm are also shown. Genes reported in bold are not associated with leukemias according to DisGeNET database (http://www.disgenet.org/), MEDline database and Genome Web Browser Ensembl.

### KCTD15 is primarily expressed in B-ALL with MLL gene rearrangement

**Figure 4:**
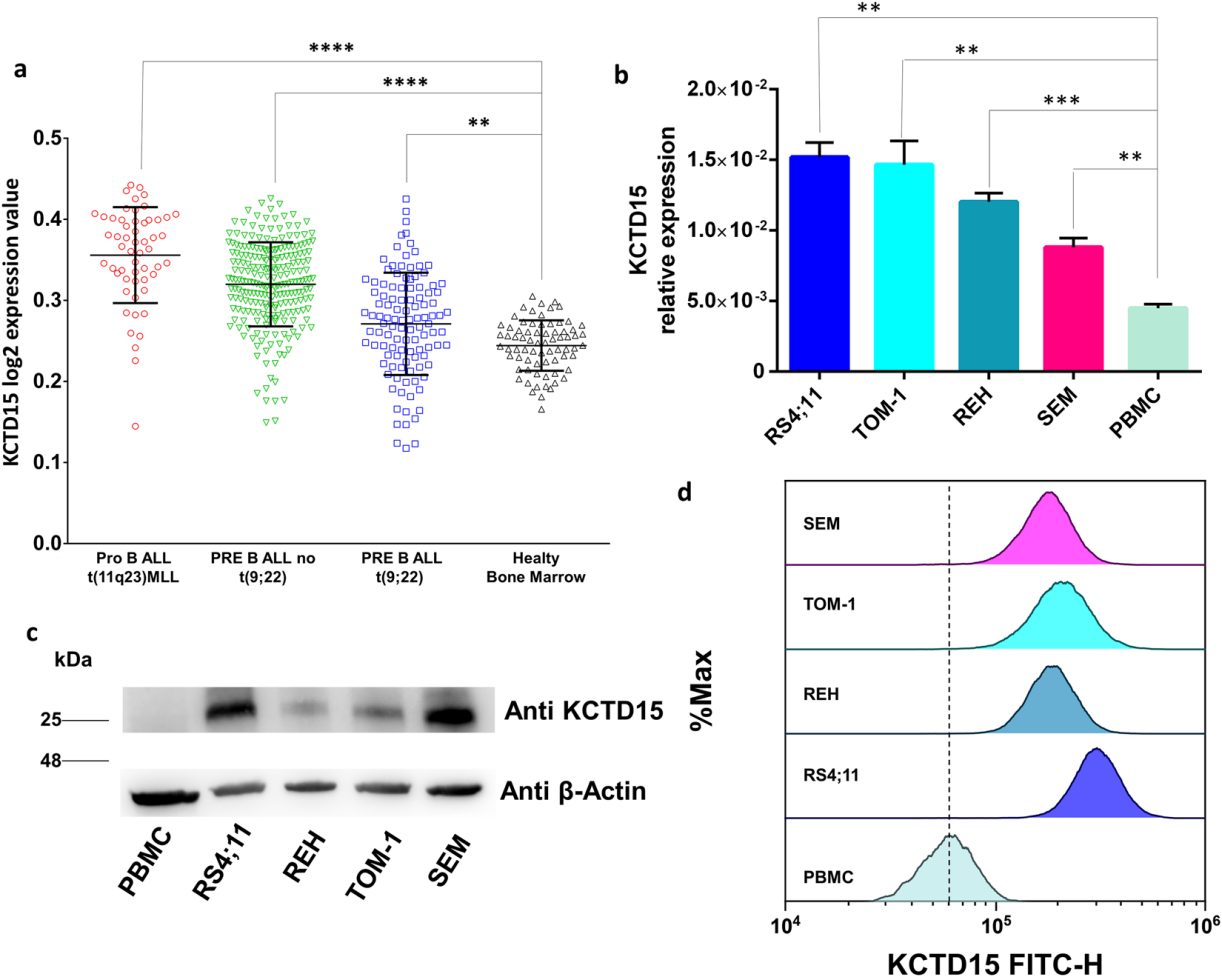
KCTD15 up-regulation in MILE study dataset (accession number GEO13159). Microarray data of KCTD15 are plotted as Log2 transformed expression values in Pro B ALL t(11q23)MLL (n = 55 samples, red circles), common/Pre-B-ALL without t (9;22) (n = 232 samples, green triangles), PRE B ALL t(9;22) (n = 111 samples, blue squares) and Healthy bone marrow (n = 72 samples, black triangles). ****p < 0.0001. **p < 0.01(Anova with Tukey’s multiple comparison test). (**b**) KCTD15 mRNA transcription levels in RS4;11, REH, TOM1 and SEM B-ALL *in vitro* model systems compared to Peripheral Blood Mononuclear Cells (PBMC). **p-value < 0,01. ***p-value < 0,001 (unpaired t-test). The relative expression was determined using the 2^−ΔCt^ method. KCTD15 relative expression is shown as mean +/− SD of four technical independent experiments. (**c**) Western blot of KCTD15 protein levels, in human B-ALL *in vitro* model systems (RS4;11, REH, TOM-1, SEM) as well as PBMC. Numbers represent the molecular weight of the protein marker expressed in kDa. (**d**) Cytofluorimetric analyses of KCTD15 protein levels, in human B-ALL RS4;11, REH, TOM-1, and SEM cell lines and PBMC.

To further validate these results in an independent study population we decided to exploit the MILE study dataset (accession number GEO13159)^20,21^. To this aim, we selected 3 subpopulations of B-ALL samples classified according to their primary genetic alterations as: proB-ALL with MLL rearrangement t(11q23) (n = 55); preB-ALL without t(9;22) (n = 232); preB-ALL with t(9;22) (n = 111). The *KCTD15* expression levels detected in these subgroups were then compared to those recorded in BM samples of healthy subjects (n = 72). Notably, in all cases the mean *KCTD15* expression levels were significantly (Tukey multiple comparison tests) higher than that observed in BM samples (Fig. 4a) and, generally, higher values were observed in cases with MLL rearrangements.

### KCTD15 expression in human B-ALL derived cell lines

To further investigate this KCTD15 deregulation in B cell leukemia, we extended our analyses to three human continuous cell lines derived from B-cell acute leukemia (RS4;11, REH, TOM-1 and SEM). All tested leukemic cell lines exhibited increased levels of *KCTD15* mRNA compared to Peripheral Blood Mononuclear Cells (PBMC) from unaffected subjects, although with a different statistical significance (Fig. 4b). The overexpression of KCTD15 was even more evident at the protein level as shown by both western blot analyses (WB) (Fig. 4c) and FCM (Fig. 4d). It is worth mentioning that, according to WB analysis, KCTD15 is highly overexpressed in the RS4;11 (Fig. 4c and Supplementary Fig. 5) and SEM cell line (Fig. 4c) that are two model systems featured by MLL rearrangement due to the t(4;11) translocation and MLL-AF4 fusion protein. For RS4;11 this observation is also confirmed by the FCM analysis (Fig. 4d); therefore, we decided to use this cell line as the *in vitro* model system for the following functional experiments.

### KCTD15 silencing induced cell death in RS4;11 cells

**Figure 5:**
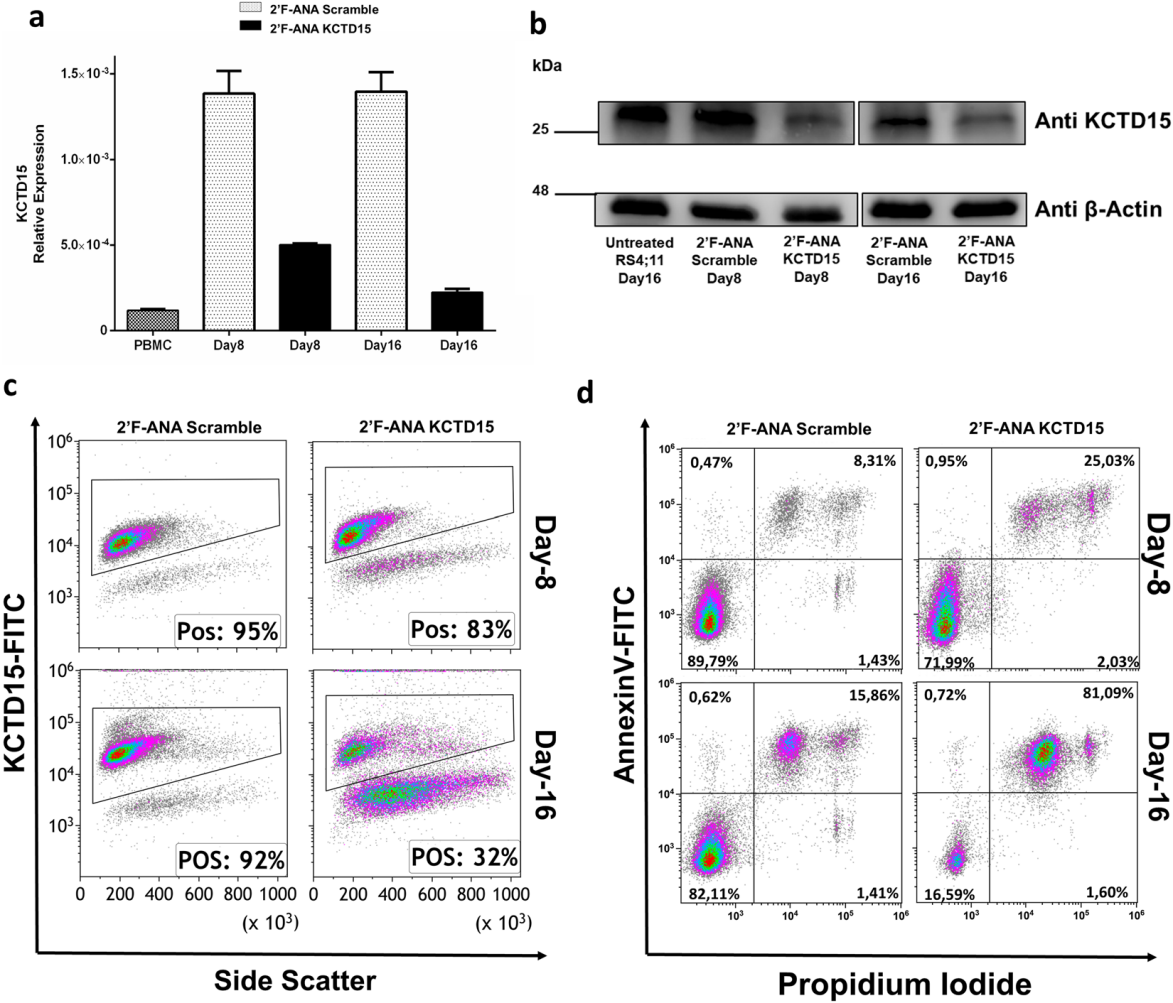
KCTD15 silencing causes leukemic cell death. (**a**) Bar-plot showing downregulation of *KCTD15* mRNA levels in 2′F-ANA KCTD15 treated cells (black bar) compared to 2′F-ANA Scramble (grey dotted-bar) at day +8 and +16 and PBMC (black dotted bar). The relative expression was determined using the 2^−ΔCt^ method and it is shown as mean +/− SD of two technical independent experiments. (**b**) Western Blot analysis of KCTD15 protein in untreated RS4;11 and 2′F-ANA Scramble and 2′F-ANA KCTD15 treated cells at day +8 and +16. Numbers represent the molecular weight of the protein marker expressed in kDa. (**c**) Time course analysis of KCTD15 intracellular levels (expressed as % of positive cells) by flow cytometry in RS4;11 cell line treated with 2′-F-ANA Scramble and 2′-F-ANA KCTD15 at day +8 and +16. (**d**) Annexin-V vs Propidium Iodide Density-plots for 2′F-ANA Scramble and 2′F-ANA KCTD15 treated RS4;11 cell lines at day 8 and 16, respectively.

To gain insights into the role played by KCTD15 in leukemic cells, the gene encoding the protein was silenced using the 2′F-ANA oligonucleotide methodology^22^ in the RS4;11 cell line (see Materials and Methods for further details). According to preliminary tests performed using a fluorescently labeled 2′F-ANA variant control (2′F-ANACtr) at a concentration of 1 µM (Supplementary Fig. 2), the transfection efficiency was ∼99.2%. The treatment of RS4;11 cells with the 2′F-ANA specific for KCTD15 (at 8 µM concentration) determined a progressive decrease of both KCTD15 mRNA and protein levels from day 8 to 16 as indicated by real-time PCR (Fig. 5a), western blot (Fig. 5b) and FCM experiments (Fig. 5c). As a consequence of *KCTD15* silencing and protein downregulation, the percentage of RS4;11 dead cells raised from 26.3% (Day-8) to ~ 80% (Day-16), while cells treated with a scrambled variant of 2′F-ANA (2′F-ANA Scramble) did not exhibit appreciable increases in the levels of mortality (Fig. 5d) when compared to the untreated counterpart (Supplementary Fig. 3).

### KCTD15 is up-regulated after PWM *in vitro* stimulation of lymphocytes

**Figure 6:**
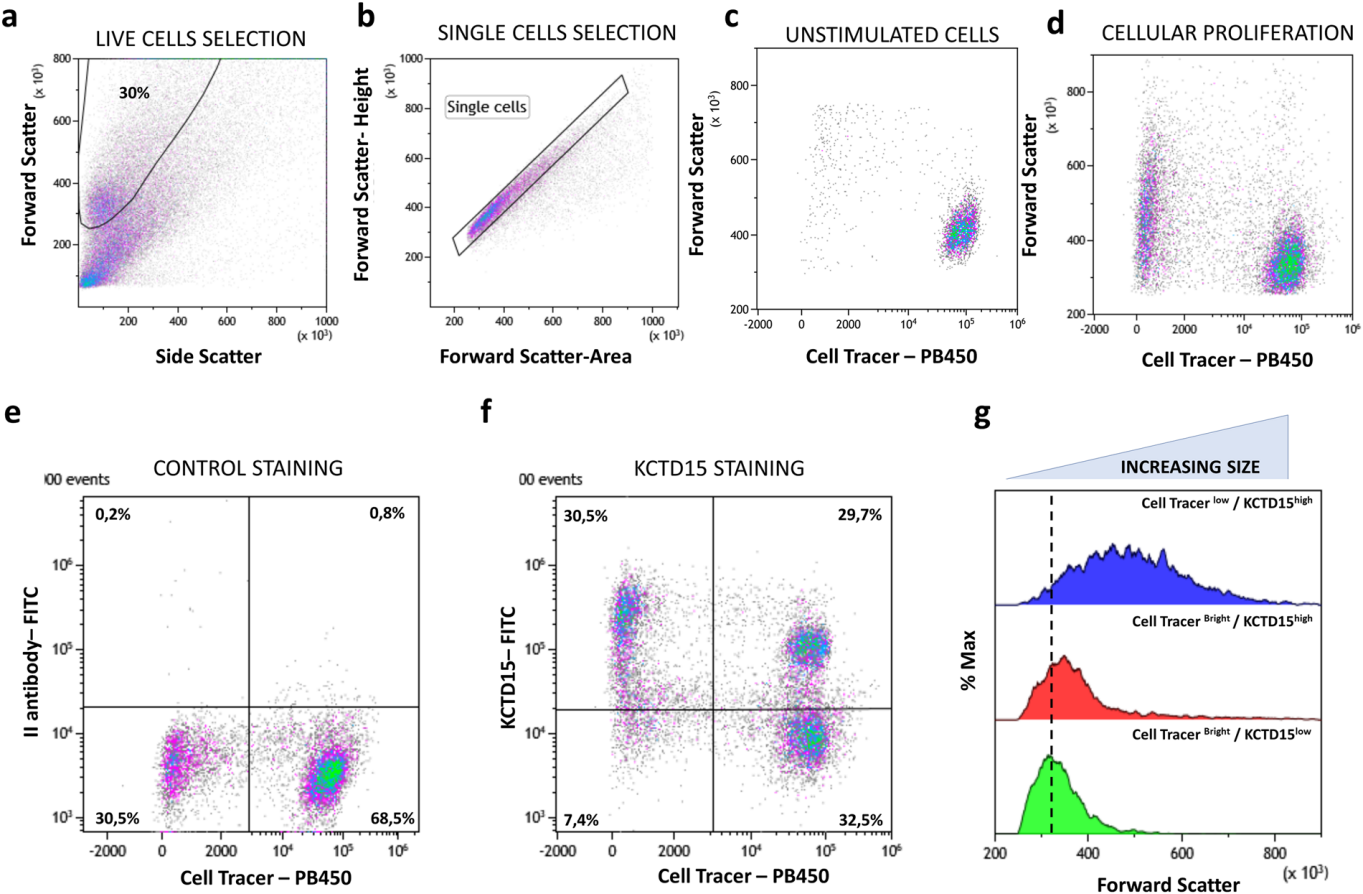
KCTD15 upregulation following to PWM stimulation. (**a**) FSC vs SSC dot-plot for the selection of live cells, the percentage is determined on the total of acquired events. (**b**) FSC-Height vs FSC-Area dot-plot used for the selection of single cells and doublets exclusion. (**c**) Dot-plot showing the FSC-height and Cell Tracer Violet intensity in unstimulated PBMC. (**d**) Dot-plot analysis of showing the FSC-height and Cell Tracer Violet intensity in PBMC stimulated for 5 days with PWM mitogen. The right side of the dot-plot shows resting cells with bright cell tracer fluorescence and low forward scatter, the left side displays active duplicating lymphocytes due to diminished cell trace fluorescence and increased FSC. (**e**) Dot-plot showing FL1 signal due to the unbound FITC conjugated secondary antibody versus cell tracer violet. (**f**) Dot-plot showing anti-KCTD15 FITC fluorescence versus cell tracer violet. (**g**) Forward scatter overlay histogram between Cell Tracer ^Bright^/KCTD15^low^ (green), Cell Tracer ^Bright^/KCTD15^high^ (red) and Cell Tracer ^low^/KCTD15^bright^ subsets. The overlay highlight the increasing FSC signal in KCTD15 cells due to PWM induced activation and proliferation. Numbers represent the percentage of gated cells.

To evaluate a possible role of KCTD15 in the proliferation of hematopoietic cells, we decided to treat the peripheral blood lymphocytes for five days with the Pokeweed Mitogen (PWM) that is able to stimulate both T and B cell growth^23,24^. To actively discriminate growing from resting cells by FCM, we stained all lymphocytes with Cell Trace Violet (CTV) before *in vitro* incubation (at day 0). After five days of continuous culture, live single cells were selected on the bases of forward (FSC) and side scatter (SSC) (Fig. 6a,b). As expected, proliferating cells presented a diminished intensity of CTV fluorescence and increased size in terms of FSC signals (Fig. 6c). In comparison to the control staining dot plot (Fig. 6d), the combined staining for KCTD15 and CTV (Fig. 6e) identified, mainly, 3 cellular subsets: (i) KCTD15^low^/CTV^high^; (ii) KCTD15^high^/CTV^high^; (iii) KCTD15^high^/CTV^low^. Notably, these last two subsets represent a cellular population of activated and proliferating lymphocytes due to the low CTV fluorescence and increasing FSC signal in comparison to the KCTD15^low^/CTV^high^ subset (Fig. 6f). This novel finding highlights the possible involvement of KCTD15 in the activation and proliferation of lymphoid cells.

Overall, the KCTD15 analysis on *in vitro* PWM activated lymphocytes confirms a possible role for KCTD15 in the malignant and normal proliferation of lymphoid cells.

## Discussion

The KCTD protein family is composed of twenty-five members (KCTD1‐21, TNFAIP1KCNRG, SHKBP1, andBTBD10) that play key actions in fundamental physio-pathological processes^11,25,26^. Studies carried out in the last decade indicate that these proteins are deeply involved in brain diseases such as medulloblastoma^12^, autism^13,27,28^, and epilepsy^29^. Moreover, these proteins are also believed to play major roles also in other processes including obesity^30,31^, breast carcinoma^32^ and scalp-ear-nipple syndrome^33,34^. According to their biochemical and functional classification, the KCTD protein family is, in general terms, subdivided into two major groups. The first one includes KCTD proteins able to interact with Culling-RING ubiquitin ligases for ubiquitination and degradation of specific substrates. The second one consists of other KCTD proteins that presumably do not interact with Cullin3 and that likely play other biochemical functions^11,12,16,35^. The inability to bind Cullin3 has hitherto been reported for KCTD proteins (KCTD8, KCTD12, KCTD12b and KCTD16) able to regulate G-protein signaling of the GABA_B_ receptor and for the homologs KCTD1/KCTD15 that are involved in diversified physio-pathological states^15,36,37^.

Sporadic relationships between leukemia and some members of the KCTD family have also been observed^38,39^. Indeed, it is that mutations of the KCTD protein SHKBP1 are linked to acute myeloid leukemia and that the haploinsufficiency of KCNRG could be associated with the progression of chronic lymphoid leukemia, although no direct connections between KCTD proteins and this pathological state have been so far reported.

Here, we show that KCTD15 is highly upregulated, both at mRNA and protein level, in B-ALL patients and related cell lines. This is particularly evident in RS4;11 cells that represent a B-ALL model system characterized by MLL rearrangement. KCTD15 downregulation by FANA silencing in this cell line induced apoptosis and cell death suggesting that this protein has a role in cellular homeostasis and proliferation. Also, the up-regulation of KCTD15 protein levels following to PWM *in vitro* stimulation highlights that the role of KCTD15 protein could be not confined to the B-ALL pathological state. This is the first time that KCTD15 has been associated with leukemia pathogenesis. KCTD15 overexpression has never been reported to be linked to pathological states, although it has been indirectly associated with obesity in several literature reports and numerous studies showed an association between this gene and obesity^31,40–42^. It is also worth mentioning that the artificial overexpression of KCTD15 inhibits the neural crest formation^37^ and that this protein is able to inhibit the Hedgehog pathway in medulloblastoma cells by increasing protein levels of the oncosuppressor KCASH2^43^.

In conclusion, the data presented in this paper show KCTD15 as a novel important player in hematology, whose up-regulation is able to sustain cell growth both in leukemic and physiological conditions. KCTD15 could be involved in still unknown signal transduction or protein degradation mechanisms that need to be defined in future studies.

## Materials and Methods

### Study population

The present study and all experimental protocols have been approved by the local ethical committee (Comitato Etico IRCCS Pascale, Naples Italy) of IRCCS-SDN with protocol number 6/16 of the 14/09/2016 and by the local ethical committee of AORN Santobono-Pausilipon (Comitato Etico Cardarelli/Pausilion, Naples Italy) with number 94 of 08/02/2017 in accordance with relevant guidelines and regulations. All participants provided informed consent signed by parents. Patients’ clinical features are presented in Table 1, patients a, b and c were selected for RNAseq experiments, while patients 1–12 for data validation. The study population was composed of 15 young patients affected by common B-cells acute lymphoid leukemia (B-ALL) consecutively admitted to A.O.R.N. Santobono-Pausilipon Hospital of Naples (Italy). At diagnosis, a bone marrow aspiration was performed to evaluate disease characteristics such as blasts morphology, immunophenotype, cytogenetic and molecular abnormalities as reported in Table 1. B-ALL was at first diagnosed in 14 patients (one case showed mixed phenotype acute leukemia characteristics), while one patient presented a pro-B-ALL. All these patients, after the completion of diagnostic procedures, started AIEOP BFM LAL 2009 protocol (ClinicalTrials.gov Identifier: NCT01117441) or subsequent guidelines (AIEOP BFM LAL 2017). All patients, except PA-9, showed a good response to treatments, with a good prednisone response on day 8 and a number of blasts < 10% at Bone Marrow Aspiration (BMA) on day 15. Interestingly, for PA-9 case it was possible to detect the Philadelphia-like associated fusion gene EBF1-PDGFRβ that allowed us to introduce the second-generation tyrosine kinase inhibitor, Dasatinib (Bristol-Myers Squibb, NY, USA) into the therapeutic plan.

### RNA sequencing

Total RNA was extracted from leukemic cells and purified B lymphocytes by Trizol (Life Technologies) reagent protocol, according to manufacturer instructions. RNA concentration and quality were determined using NanoDrop ND-1000 (ThermoFisher Scientific, MA, USA) spectrophotometer and Tapestation 4200 (Agilent Technologies, CA, USA) respectively. Indexed libraries were prepared from 150–200 ng of purified RNA with TruSeq Stranded total RNA Sample Prep Kit (Illumina, CA, USA) according to the manufacturer’s instructions. Libraries were quantified using the Tapestation 4200 and pooled such that each index-tagged sample was present in equimolar amounts, with final concentration of the pooled samples of 2 nM. The pooled samples were subjected to cluster generation and sequencing using an Illumina NextSeq. 500 System in a 2 × 100 paired-end format at a final concentration of 1.8pmol. For RNA-seq analysis see Supplemental Methods.

### Statistical analysis for RNA-seq data

Statistical analysis for RNA-seq data and GEO data set processing, was carried by R software (version 3.4.3). CPM criteria were applied for RNA-seq raw data matrix filtered for low counts. Normalization step was performed by applying the upper quartile (UQUA) approach. To estimate differentially expressed genes (DE) we considered p-adj ≤ 0.01 for DEseq. 2 method and a posterior probability ≥ 0.99 for NOISeqBio method. We considered only 873 DE genes with |log2FC| ≥ 1. To generate volcano plot we filtered out genes with undefined estimated p-value (NA), in total 109 genes out of the 17085 expressed genes using R package graphics. Microarray data from Microarray Innovations in Leukemia (MILE study stage I data) were downloaded from GEO database (accession number GSE13159, PMID:18573112, PMID:20406941). We considered the whole dataset, performed a full quantile normalization and then transformed in logarithmic scale the probe set expression values, using R package limma^44^. In order to investigate KCTD15 expression profile, we calculated the probe set mean value for the following BM samples: n = 232 c/pre-B-ALL not (9;22), n = 55 Pro B ALL t(11q23)MLL, n = 111 PRE B ALL t(9;22) nd n = 72 healthy BM. Scatter plot was represented, using GraphPad Prism 7. Gene-disease association in the Table 2, were obtained through the database DisGeNET^17^ and manual query with the following criteria: “gene symbol” AND “leukemia” OR “acute lymphoblastic leukemia” in the MEDline database and the Genome web browser Ensembl, section “Phenotype data”.

### Patient samples and cell lines

Bone Marrow MonoNuclear Cells (BM-MNC) were obtained by density gradient centrifugation (Pancoll® density 1,077 g/L, PanBiotech, Aidenbach, Germany) at 400xg and stored by the Biobank of SDN Institute (10.5334/ojb.26) in the vapor phase of liquid nitrogen until the use. In the case of BM aspirates form two patients, identified as PA-11 and PA-12, it was not possible to get a sufficient number of cells to perform RT-PCR due to *punctio sicca*. Normal B lymphocytes for RNAseq were attained from fresh venous blood collected in 3 mL EDTA vacutainer tubes (Becton Dickinson, CA, USA, Catalog. #367835) from three healthy volunteers. Mononuclear cells were isolated by density gradient centrifugation before applying the EasySep™ Human B Cell Enrichment Kit (Stemcell Technologies Inc. Catalog #19054) according to manufacturer instructions. In all cases about 1 × 10^6^ total live cells were obtained with a purity greater than 90% as assessed by FCM. The following authenticated human cell lines were used: RS4;11, REH, TOM-1 and SEM. All these cell lines were grown in Iscove’s Modified Dulbecco’s Medium (IMDM) supplemented with 10% Foetal Bovine Serum (GIBCO) and 1% Glutamax (Invitrogen). All cultures were incubated at 37 °C and 5% CO2 and seeded in 24 well plates or T-25 culture flasks.

### Real-time PCR

RNA from human B-ALL cell lines and BM-MNC of B-ALL patients was isolated using Trizol Reagent protocol (Thermo Fischer Scientific). cDNAs synthesis were performed using SuperScriptTM III First-Strand Synthesis SuperMix kit (Thermo Fisher) according to the manufacturer instructions. The cDNA concentrations were evaluated using QubitTM 4 Fluorometer (Thermo Fischer Scientific, Massachusetts, USA). RPS18 gene was used as housekeeping. Oligonucleotides used for RT PCR are: KCTD15fw = 5′-TGTCATGGCAACAGAACGTG-3′; KCTD15rev = 5′-CAGAGATCCCACCGCTGTAT-3′; RPS18fw = 5′-CGATGGGCGGCGGAAAATA-3′; RPS18rev = 5′-CTGCTTTCCTCAACACCACA-3′.

RT-PCR experiments were performed using C1000 Touch Thermal Cycler (Bio-Rad, CA, USA) using iQ SYBR Green Supermix (1708882, Bio-Rad) applying for the following program: initial denaturation (95 °C, 3 min), 40 cycles of denaturation (95 °C, 10 sec), annealing (60 °C, 30 sec) and elongation (72 °C, 30 sec), final elongation (72 °C, 10 min) and a final hold (4 °C). The melting curve was generated in the range of 60–95 °C. The reaction volume was 25 µl. Each reaction was performed in duplicate. Samples were normalized to their RPS18 level using the 2^−ΔCt^ method. Two independent experiments were performed for each RT-PCR. Data were analyzed using Biorad CFX Maestro version 1.0 (Bio-Rad).

### Western blot assays

Lysates from human B-ALL cell lines and PBMC (50 μg of protein extracts) were analyzed by Western Blot to check the expression of the proteins. Antibodies used were: anti KCTD15 (GTX50002, Genetex International, USA), and anti β-actin (ab11004, Abcam, UK) as internal control. Proteins were acquired using ChemiDoc Imaging system (Biorad, USA) coupled with Image Lab software.

### Fluorescence microscopy

BM smears of B-ALL patients were air-dried overnight and then fixed at −80 °C for 15 minutes with a −80 °C pre-cooled solution of Methanol-Acetone 1:1 (v/v). Subsequently, the slides were subjected to blocking with solution of 3% (w/v) BSA in PBS pH 7.4 at Room Temperature (RT). Anti KCTD15 monoclonal antibody was diluted 1:100 in a solution of PBS + 1% (w/v) BSA and then all slides were incubated for 4 h at + 4 °C. After three wash steps in PBS for 5 minutes each, FITC- conjugated anti-mouse secondary antibody (ab7064, Abcam, UK) diluted 1:200 in a solution of PBS + 1% (w/v) BSA was incubated for 1 h at 4 °C in the dark. After additional three wash steps in PBS, a solution of 4′,6-Diamidino-2-Phenylindole, Dihydrochloride (DAPI, Thermo Fischer Scientific D1306) diluted 1:35000 in PBS was used for coloring of the nuclei.

Images were obtained using an automated upright microscope system (Leica DM5500 B) coupled with Leica Cytovision software.

### Flow cytometry experiments

Flow cytometry experiments with a minimum of 10.000 recorded events were performed using the Cytomics FC500 except in case of Cell Trace Staining on stimulated lymphocytes where the Cytoflex V2-B4-R2 (Beckman-Coulter, CA, USA) instrument was used. Routinely control of instrument sensitivity was performed and no change in instrument sensitivity throughout the study was seen. Intracellular or combined intracellular plus surface staining was performed by the use of PerFix Expose kit (B26976, Beckman Coulter,) according to manufacturer instruction. Data were analyzed using Kaluza analysis software version 2.1 (Beckman-Coulter, CA, USA). The following monoclonal antibodies were used for FCM experiments: anti KCTD15 (GTX50002, Genetex International, USA), CD19 (CD19-RD1, 6603024 Beckman Coulter) and CD45 (CD45-PC5, IM2652U Beckman Coulter). FITC- conjugated anti mouse secondary antibody (ab7064, Abcam, UK) was used for anti-KCTD15 detection.

### Proliferation assay

For proliferation assays, PBMC, derived from a healthy male with 39 years old, were stained with a solution of 5 μM of CellTrace^TM^ Proliferation Kits (C34557, ThermoFisher Scientific, USA) in PBS 1x for 20 min according to manufacturer instructions. After the incubation time, the free dye was removed diluting five times the original staining volume with complete culture medium (IMDM with 10%FBS and 1%L-Glutammine). After centrifugation, cells were cultured for 5 days in culture medium supplemented with 2.5 μg/mL of PWM mitogen used to activate lymphocytes. Cell growth and viability were routinely checked by contrast microscope observation. At the end of the culture period, all cells were harvested and centrifugated for the PerFix Expose protocol.

### KCTD15 silencing by 2′-Deoxy, 2′Fluroarabino Nucleic Acids (2′F-ANAs) Oligonucleotides

For KCTD15 silencing we used 2′-deoxy-2′-fluoro-beta-D-arabinonucleic acid (2′F-ANA) modified oligonucleotides (ASOs)^22,45^. In our case RS4;11 cells were seeded in complete media at a concentration of 5 × 10^5^ cells/ml, and two different concentrations (4 and 8 µM) of 2′F-ANAs against KCTD15 were tested. The antisense oligonucleotide sequence for KCTD15 that we selected was: AACCTATCAAGTTTGTCCAGC (5′ – 3′ orientation). At the same time, 1 µM of fluorescently labeled control 2′F-ANA was used to check the transfection efficiency (Supplementary Fig. 2). Before FCM acquisition cells were washed twice with DPBS supplemented with 2% FBS for removing fluorescent oligos stuck to the membrane. According to the findings of Mayumi Takahashi *et al*.^46^, we confirmed that FANA oligos penetrate into the cells very easily. Then, for our experiments, a concentration of 8 µM of 2′F-ANA was chosen. Cells were harvested at different time points of incubation (up to 16 days) to check KCTD15, expression as described. Considering the long incubation time for optimal KCTD15 silencing in RS4,11 cells, we split the cells and added fresh 2′F-ANA oligos to the culture media. Cell viability was also evaluated using ANNEXIN V – FITC Kit - Apoptosis Detection Kit (IM3546, Beckman Coulter, USA).

### Statistical analysis and reproducibility

P-values were calculated as described in individual figure legends using Graphpad Prism 7 (Graphpad Software). Numbers of biological and/or technical replicates, as well as a description of the statistical parameters, are stated in the figure legends. All experimental images are representative of at least two independent experiments. No statistical method was used to predetermine sample size and experiments were not randomized. Statistical analysis for RNA-seq experiments is reported in Supplemental Methods.

## Supplementary information

Supplementary Information.
