# Supplementary material for: KCTD15 is overexpressed in human childhood B-cell acute lymphoid leukemia
Source: Sci Rep. 2019 Dec 27;9:20108. doi: 10.1038/s41598-019-56701-7 (PMC6934626; doi:10.1038/s41598-019-56701-7)
Supplement: Supplementary file 1 — Supplementary Information. [file 41598_2019_56701_MOESM1_ESM.pdf]

TITLE PAGE

Article Title: KCTD15 is overexpressed in human childhood B-cell acute lymphoid leukemia

Authors: Giovanni Smaldone[1], Giuliana Beneduce[1,4], Mariarosaria Incoronato[1], Katia Pane[1], Monica Franzese[1], Luigi Coppola[1], Angela Cordella[1,2], Rosanna Parasole[4], Mimmo Ripaldi[4], Giovanni Nassa[2], Andrea Soricelli[1,5], Luigi Vitagliano[3*], Peppino Mirabelli[1*] and Marco Salvatore[1]

Affiliations:

[1] IRCCS SDN, Via E. Gianturco 113, 80143, Napoli, Italy.

[2] Laboratory of Molecular Medicine and Genomics, Department of Medicine and Surgery, University of Salerno, Baronissi, Italy.

[3] Institute of Biostructures and Bioimaging, C.N.R., 80134, Napoli, Italy

[4]Department of Pediatric Hematology-Oncology, Santobono-Pausilipon Hospital , Naples , Italy.

[5]Department of Sport Sciences & Healthiness, University of Naples 'Parthenope', 80131, Napoli, Italy

*** Corresponding Authors**

-Dr. Peppino Mirabelli, PhD IRCCS SDN, Napoli Via Emanuele Gianturco, n°113 80143 – Naples Italy Mail: pmirabelli@sdn-napoli.it

-Dr. Luigi Vitagliano, PhD Institute of Biostructures and Bioimaging, C.N.R. Via Mezzocannone, n°16 80134 – Naples Italy Mail: luigi.vitagliano@unina.it

Supplementary Fig. 1

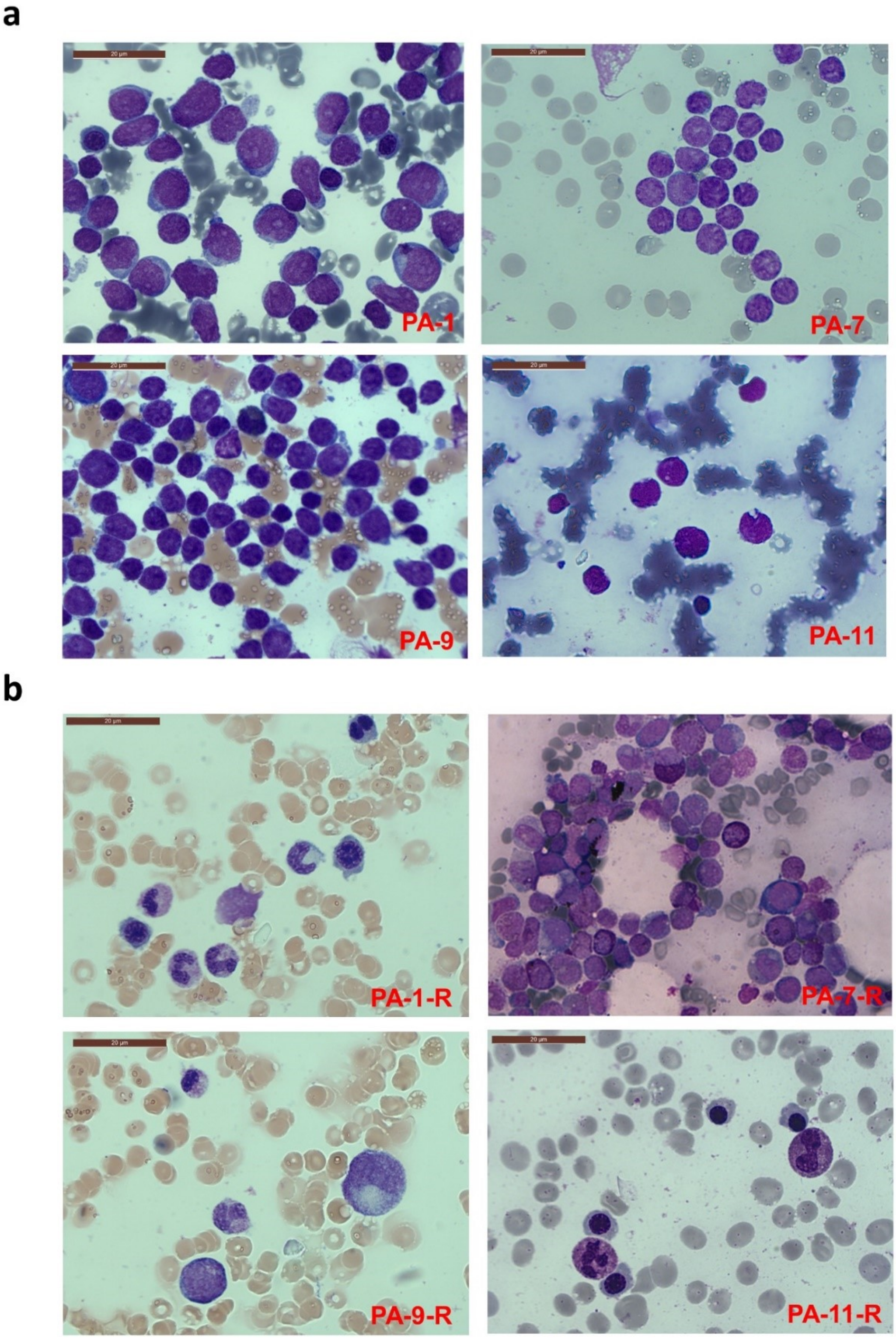

Supplementary Fig.1: Morphological examination of bone marrow blood smear. **a** – BM smears of four pediatric patients affected by acute lymphoblastic leukemia. The presence of blasts is evident in each patient. **b -** BM smears from the same patients after treatment; the smears show myeloid and erythroid elements in a different stage of maturation in the absence of lymphoid blasts. PA-#= patient # at diagnosis. PA-#-R= patient # after treatment at day +33. Magnification 63x. Scale bars 20µm.

Supplementary Fig. 2

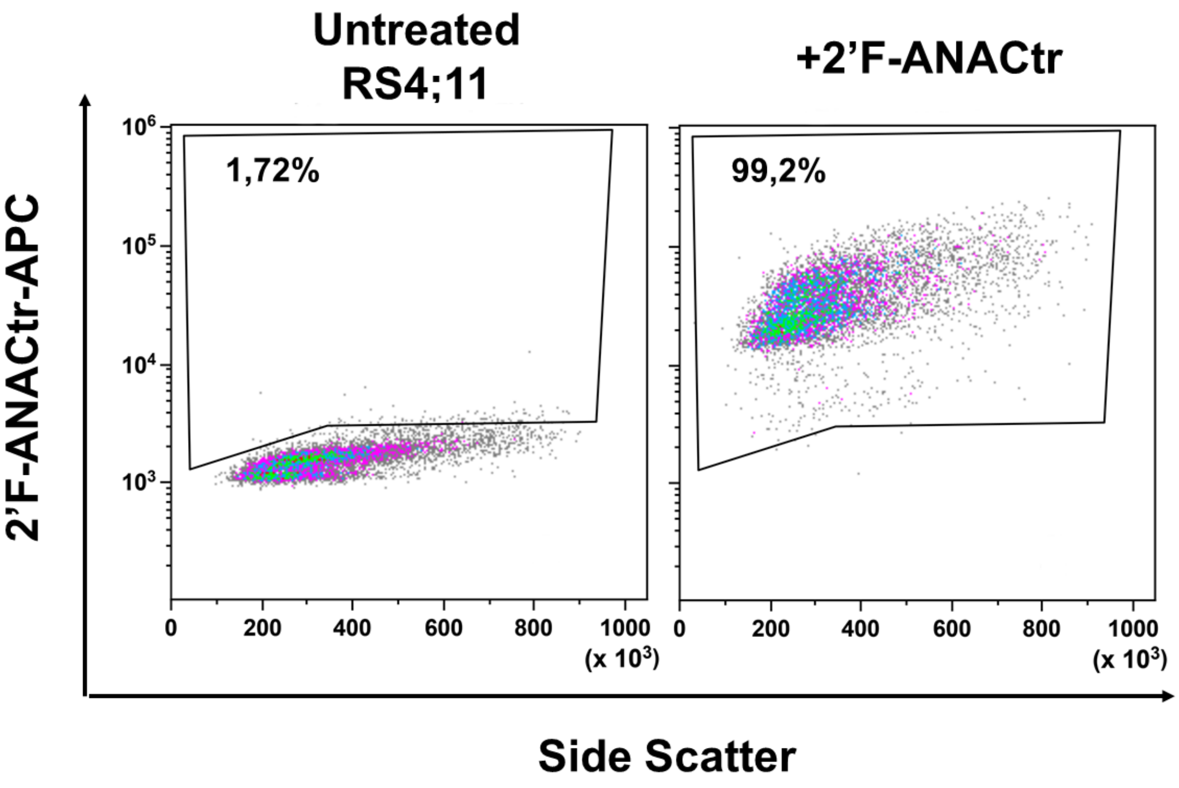

Supplementary Fig. 2: **2’F-ANA ctr internalization**. Capability of 2’F-ANA red control oligonucleotide (2’F-ANACtr) to be internalized in 99.2% (dot-plot on the right) of RS4;11cells after 24h of incubation without the use of electroporation or liposome mixture.

Supplementary Figure 3

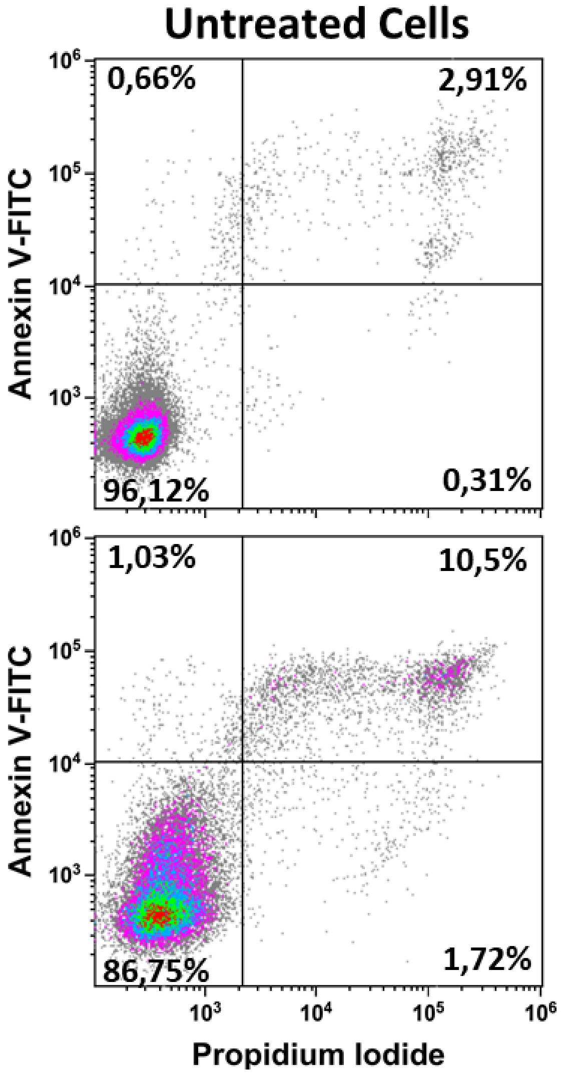

Supplementary Figure 3: Untreated RS4;11 viability. Annexin-V vs Propidium Iodide Density-plots for untreated RS4;11 cell line at day 8 (upper panel) and 16 (lower panel). Numbers represent the percentage of gated cells.

Supplementary Figure 4

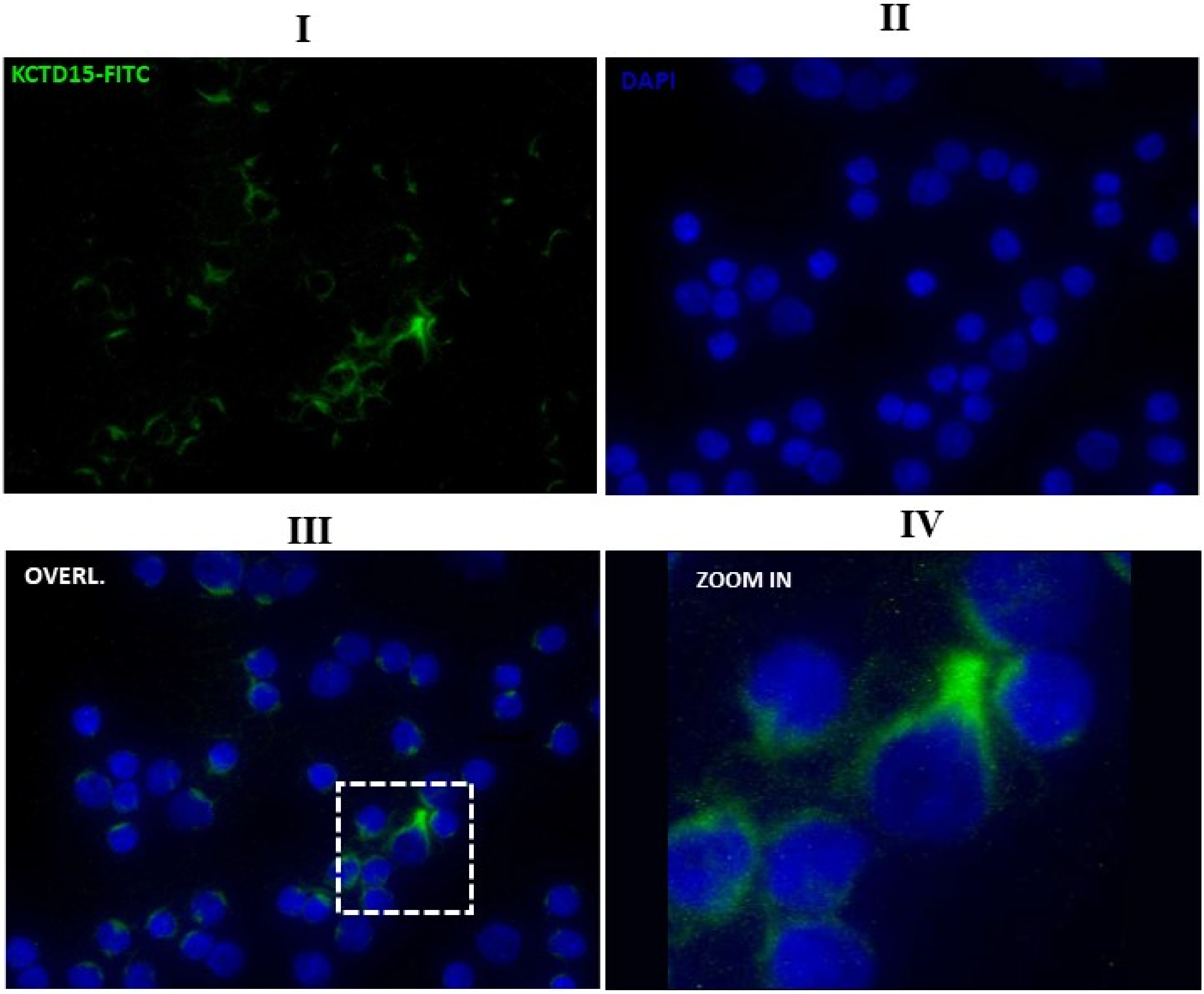

Supplementary Figure 4: KCTD15 fluorescence in trypsinized BM smear. KCTD15 endogenous fluorescence was determined in BM smear treated with trypsin for removing unwanted fluorescence related to possible protein crosslinks caused by the fixation protocol. Endogenous KCTD15 was labeled with FITC-conjugated secondary antibody. Column I) KCTD15-FITC fluorescence (green). Column II) Nuclei staining with DAPI (blue). III) Overlapping of FITC and DAPI channels. IV) Enlarged detail of overlapped channels. According to figure 2C, KCTD15 fluorescence is mainly localized in the cytoplasm of leukemic blasts. The zoom-in panel confirms the cytosolic localization showing KCTD15 fluorescence in a cytosolic extension of a leukemic blast. Magnification 63x.

Supplementary Figure 5

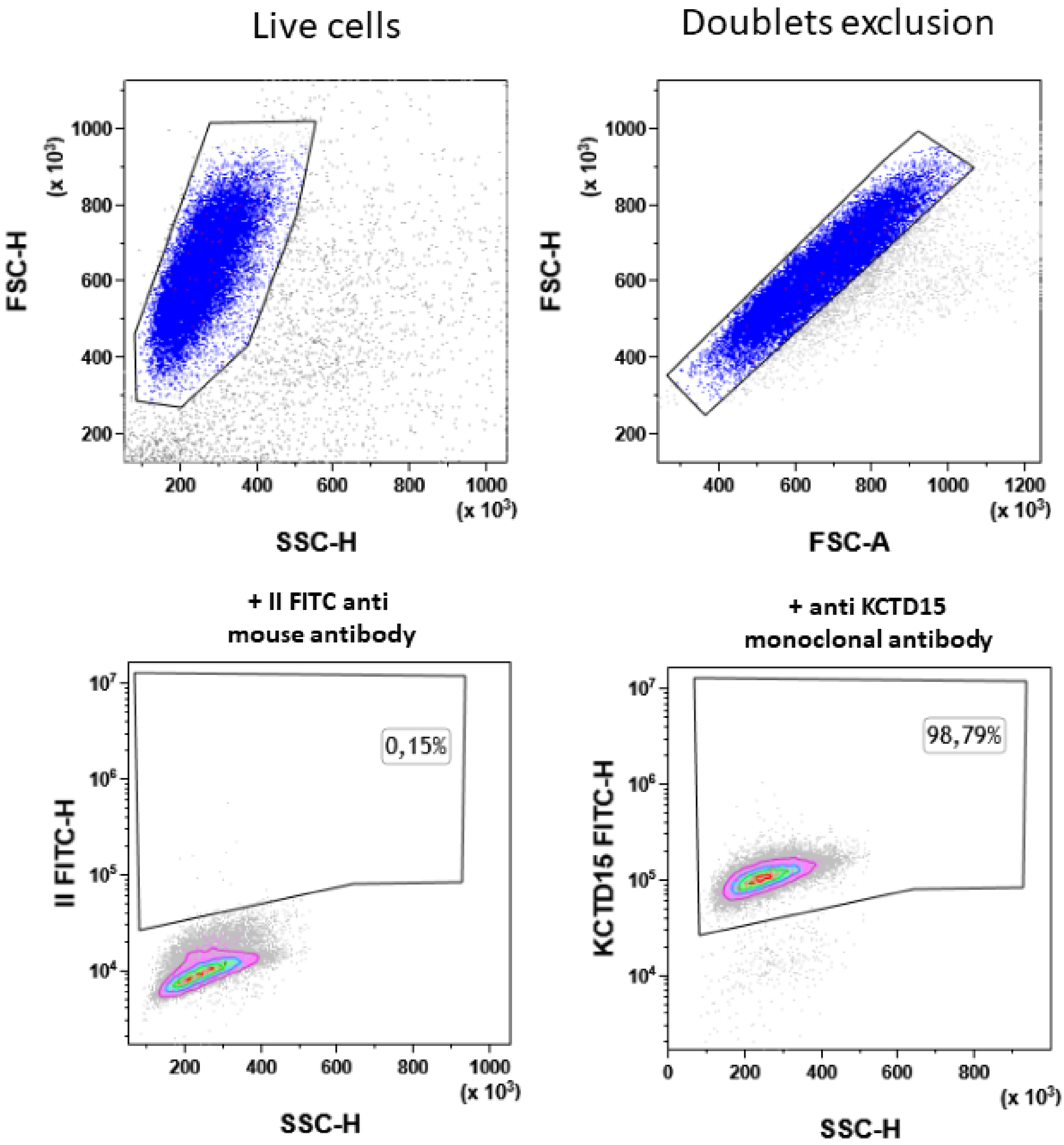

Supplementary Figure 5: RS4;11 Intracellular KCTD15 fluorescence by flow cytometry after permeabilization and trypsinization. The upper panel left and right shows the selection of live cells and doublets exclusion, respectively. The lower panel left shows FITC fluorescence for FITC-conjugated anti-mouse secondary antibody only. Lower panel right displays the KCTD15 detection using anti KCTD15 mouse monoclonal antibody and secondary anti-mouse FITC conjugated antibody. Before staining with detection antibodies, fixed RS4;11 cells were treated with trypsin 0.25% in HBSS w/o Ca+2 and Mg+2.

Original image for western blot from Figure 4c

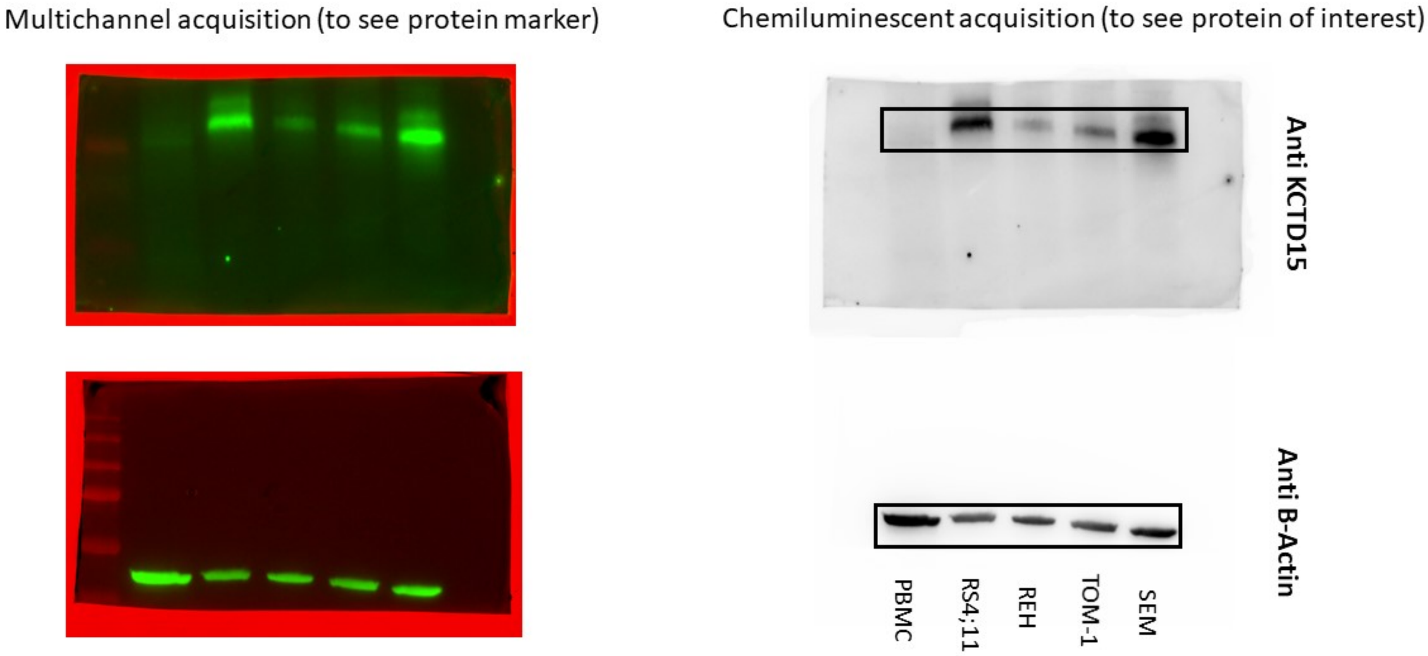

This figure reports the full-length blots used for Figure 4c. On the left multichannel acquisition to see protein marker. On the right chemiluminescent acquisition to see protein of interest. The acquisition was performed using the ChemiDoc Imaging system (Biorad, USA) coupled with Image Lab software. Black squares represent the image reported in the manuscript.

Original image for western blot from Figure 5b

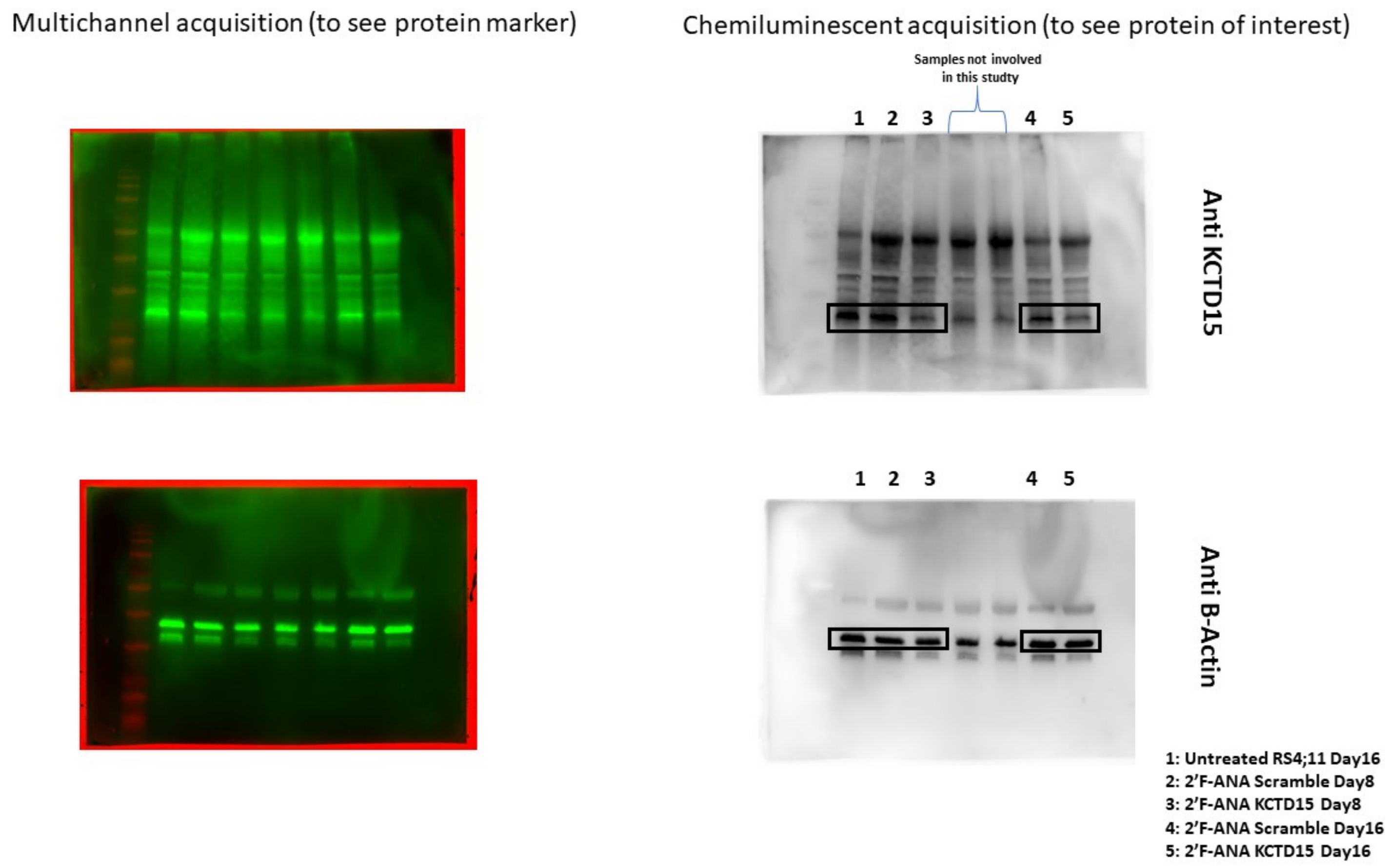

This figure reports the full-length blots used for Figure 5b. Chemiluminescent acquisition to see protein of interest was reported. The acquisition was performed using the ChemiDoc Imaging system (Biorad, USA) coupled with Image Lab software. Black squares represent the image reported in the manuscript.
